# Prognostic Role of Serum Adrenomedullin in Patients with Ventilator Associated Pneumonia

**DOI:** 10.3390/arm90040044

**Published:** 2022-08-18

**Authors:** Tamer Abdallah Helmy, Haitham Hamdy Tammam, Michael Ebrahim Leuis, Bassem Nashaat Beshey

**Affiliations:** Critical Care Medicine Department, Faculty of Medicine, Alexandria University, Alexandria 5424010, Egypt

**Keywords:** ventilator associated pneumonia, adrenomedullin, prolonged mechanical ventilation, sepsis, septic shock, mortality

## Abstract

**Highlights:**

- Ventilator associated pneumonia (VAP) is a common type of sepsis in mechanically ventilated patients that can progress to septic shock and increased mortality.
- Adrenomedullin (ADM) was found to be elevated in septic patients, so we explored the possible prognostic role of adrenomedullin in VAP patients.
- A total of 140 mechanically ventilated patients with proven VAP after medical ICU admission were consecutively enrolled into this prospective observational study.
- Serum ADM on enrollment could predict the unfavorable outcome with a sensitivity of 96.25%. After 5 days, it showed a sensitivity of 57.5% and a specificity of 100%.
- We found that serum adrenomedullin when measured at days 0 and 5 of VAP diagnosis may serve as an early predictor of unfavorable outcome (prolonged mechanical ventilation, septic shock, and mortality).

**Abstract:**

Objective: Ventilator associated pneumonia is a common type of sepsis that occurs to about 9–27% of all mechanically ventilated patients and 20–50% of them develop septic shock. Several clinical, laboratory, and radiological methods have been used for diagnosing VAP. Adrenomedullin (ADM) has been found to be elevated in the plasma of septic patients. The study aim was to explore the prognostic role of ADM in the VAP patients. Design: A prospective observational study. Setting: Intensive Care Department of Alexandria University Hospitals. Patients: A total of 140 patients with proven VAP after medical ICU admission were consecutively enrolled. Methods: APACHE II score, SOFA score, CRP, lactate, and serum ADM were measured at day 0 of VAP diagnosis and 5 days later. The results were correlated with the outcomes of patients. Results: APACHE II, lactate, and serum ADM on day 0 could predict an unfavorable outcome. ADM prediction power was significantly higher than APACHE II and lactate. Day 5 readings of all tested parameters could predict occurrence of the unfavorable outcome. ADM on day 0 showed the highest sensitivity (96.25%). Conclusions: Serum adrenomedullin when measured at days 0 and 5 of VAP diagnosis may serve as an early predictor of unfavorable outcome.

## 1. Introduction

Ventilator-associated pneumonia (VAP) as a type of sepsis in the intensive care unit (ICU) could be defined as pneumonia that occurs 48–72 h or later following endotracheal intubation contributing to about 50% of hospital-acquired pneumonia and is estimated to occur in 9–27% of all mechanically ventilated patients [1,2,3]. It is the second most common nosocomial infection in the ICU and is associated with increased days of ICU stay and mechanical ventilation [4]. From the clinical point of view, effective prophylaxis, early diagnosis, and proper treatment are the most important topics in VAP management. Despite this, nearly 50% of VAP patients develop septic shock [5].

Due to difficulties and disparities in diagnosing VAP, several clinical, laboratory, and radiological methods have been recommended, but none has the needed sensitivity or specificity to accurately identify this disease. The Clinical Pulmonary Infection Score (CPIS) considers the combined clinical, physiological, microbiological, and radiographic evidence to allow the numerical value to predict the presence or absence of VAP [6].

Adrenomedullin (ADM) behaves like a hormone and is produced by multiple tissues during physiologic and infectious stress with varying physiological functions including vasodilator, anti-inflammatory, and antimicrobial activity. Serum ADM was found to be elevated in patients with sepsis and septic shock and could serve as a diagnostic and prognostic marker in septic patients [7,8]. Hence, we had the idea to search for a possible prognostic role for serum ADM in VAP patients if compared to other diagnostic and/or prognostic markers. The outcome of the studied patients was classified into favorable outcome (successful extubation) or unfavorable outcome (prolonged mechanical ventilation, development of septic shock, and 28-days mortality). Our study aim was to explore the possible prognostic role of adrenomedullin on outcome in VAP patients.

## 2. Patients and Methods

This prospective observational study was carried out on one hundred and forty (140) patients, according to the sample size calculation. For the estimation of the minimum sample size required, data from previous literature were used to calculate the effect size. As none of them compared patients with favorable and unfavorable outcomes, studies that had assessed the prognostic role of ADM in predicting septic shock and/or overall mortality among VAP patients were used [9,10]. This yielded a minimum sample of 24 patients to achieve a 90% power at a 5% level of significance. To allow for multiple comparisons, the sample was increased to 140 patients. The sample size was calculated using G*Power 3.1.9.4 statistical software (Heinrich Heine University Dusseldorf; NorthRhine-Westphalia; Germany).

Patients were admitted to the medical Intensive Care Units of the Alexandria University Hospitals during the period from 1 June 2016 until 31 December 2017. Patients were consecutively included into the study as having VAP if they were intubated and ventilated for ≥48 h and had a radiographic infiltrate that was new or progressive, with clinical findings suggesting infection. Clinical findings included new onset fever, purulent sputum, leukocytosis (or leukopenia), and decline in oxygenation [1]. Modified clinical pulmonary infection score (CPIS) was calculated for these patients and were included when the CPIS was >6 [6].

Patients under 18 years old, those with chronic renal or liver disease, conditions influencing adrenomedullin level (chronic heart failure and acute coronary syndrome, acquired immune deficiency syndrome malignancy, and immunosuppressive status) were excluded from the study [8]. Approval of the Medical Ethics Committee of Alexandria Faculty of Medicine was obtained. An informed written consent was taken from the patients’ next of kin before their enrollment in the study.

Upon enrollment into the study, patients were subjected to the collection of demographic data (age and sex), associated co-morbidities, main diagnosis, duration of intubation and ventilation before diagnosis of VAP, routine ICU investigations (including those needed for calculation of APACHI II score, SOFA score, and modified CPIS), and endotracheal aspirate with semiquantitative cultures. Acute Physiology and Chronic Health Evaluation II (APACHE II) score calculation [11], Sequential Organ Failure Assessment (SOFA) score [12], serum C-reactive protein (CRP) (mg/dL), serum lactate (mg/dL), and serum adrenomedullin (pg./mL) were measured at different intervals, namely at day-0 (day of enrollment) and at day-5 (5 days after diagnosis of VAP). ADM was measured using the enzyme-linked immunosorbent assay (ELISA) using a double antibody sandwich method. The ELISA assay included a purified human anti-AM antibody as the capture antibody, and perborate/3, 3, 5, 5-tetramethylbenzidine as the substrate (Sphingotec, GmbH, Penzberg, Germany) [10,13].

All patients were managed in the ICU according to the guidelines of managing VAP [14]. The outcome was either a favorable outcome (successful extubation) or unfavorable outcome (prolonged mechanical ventilation, development of septic shock, and 28-days mortality). Survival was recorded daily by in-hospital observation and later by a phone call. Prolonged mechanical ventilation was considered if >10 days’ duration after VAP diagnosis. Septic shock was defined as the presence of sepsis induced organ dysfunction accompanied by a sustained arterial hypotension (mean arterial pressure <65 mmHg) requiring vasopressor therapy despite adequate volume resuscitation [15]. The role of ADM as one of the possible biomarkers in the prognosis of VAP was evaluated in conjunction with some other parameters; namely, APACHE II score, SOFA score, lactate, and CRP, hoping to find out which of them could predict the incidence of unfavorable outcome (prolonged MV, septic shock, and mortality) at different intervals (namely days 0 and 5 of VAP diagnosis).

Data were analyzed using IBM SPSS version 20 (IBM Corp, Armonk, NY, USA). The Kolmogorov–Smirnov test was used to assess the normality of the quantitative variables. Normally distributed variables were presented as the mean and standard deviation, however, the median and interquartile range (IQR) was used for the skewed data. Groups were compared regarding the qualitative variables using the Chi-square test. For the quantitative variables, a comparison was conducted using one-way ANOVA or the Kruskal–Wallis test according to the distribution of variables. The correlation between the variables was conducted using the Spearman or Pearson correlation. Analysis was conducted at a 5% level of significance. The receiver operating characteristic (ROC) curve was constructed, and the area under the ROC curve (AUC) was determined to assess the predictive value of ADM and other predictors in the occurrence of unfavorable outcome at two points of time (at day 0 of VAP, and 5 days later) with the calculation of then area under the curve (AUC) as well as the optimal threshold with the best predictive ability with its associated sensitivity, specificity, and predictive values.

## 3. Results

**Figure 1:**
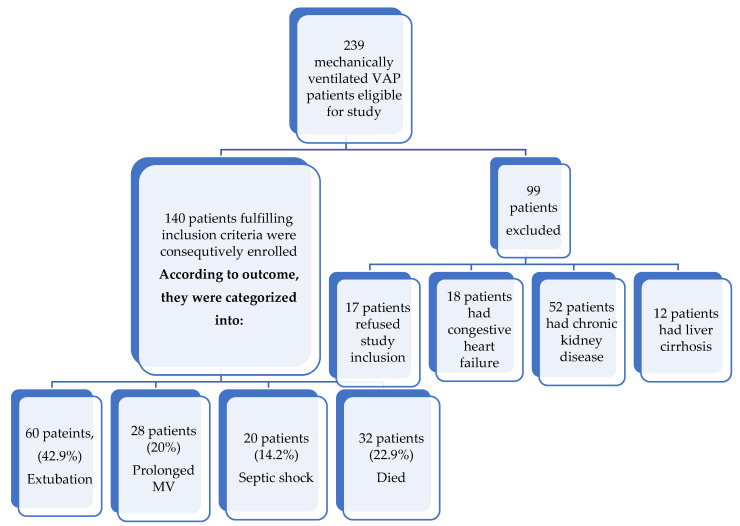
The patients’ flow chart. MV-Mechanical ventilation.

Figure 1 shows the patients’ flow charts. A total of 239 mechanically ventilated VAP patients were eligible for this study. Ninety-nine patients were excluded due to refusal of study (17 patients), 18 patients had congestive heart failure affecting serum adrenomedullin measurement, 52 patients had chronic kidney disease, and 12 patients had liver cirrhosis. A total of 140 patients fulfilling the inclusion criteria were consecutively enrolled. According to outcome, they were categorized into four groups. Sixty patients (42.9%) were successfully extubated (representing favorable outcome). The rest of the patients represented an unfavorable outcome. Twenty-eight patients (20%) had prolonged MV, 20 patients (14.2%) developed septic shock, and 32 patients (22.9%) died.

Table 1 shows the baseline patients’ criteria at the time of enrollment. The studied patients showed no statistical significance with regard to age or sex (*p* = 0.061 and 0.555 respectively). Diabetes mellitus, hypertension, ischemic heart disease, and chronic obstructive pulmonary disease as patient comorbidities were represented in all outcome groups (*p* = 0.989). Coma, respiratory failure, sepsis, and toxidromes were the main diagnoses of the enrolled patients (*p* = 0.989). MV days before VAP diagnosis in all patients was statistically insignificant (*p* = 0.641). Methicillin resistant staphylococcus aureus (MRSA), *Pseudomonas aeruginosa*, *Klebsiella pneumoniae*, *E-coli*, *Acinetobacter*, *Candida albicans*, and contamination were the main results of the semiquantitative cultures of tracheal aspirate at the day of enrollment (*p* = 0.999).

Table 2 shows the patients’ distribution according to the different parameters at different intervals. The studied parameters were collected at two-time intervals: day zero—day of enrollment into the study after confirmed VAP diagnosis, and day five—5 days later. The APACHE II score was significantly higher in the unfavorable outcome groups at both time intervals (*p* < 0.001). The day 5 levels of CRP were significantly higher in the unfavorable outcome groups (*p* < 0.001). The serum lactate values at both days 0 and 5 were significantly higher in the unfavorable outcome groups (*p* < 0.001). The SOFA score showed significantly higher values only by day 5 in the unfavorable outcome groups (*p* < 0.001). The serum ADM values were significantly higher in the unfavorable outcome groups at both days 0 and 5 (*p* < 0.001). All variables showed variable significant differences between individual groups.

Table 3 shows the correlation between adrenomedullin and other parameters at different intervals. At day 0, adrenomedullin was significantly correlated only to the APACHE II score and lactate, while at day 5, it was significantly correlated to all of the measured parameters (CRP, SOFA score, APACHE II score, and lactate). Days of MV and ICU stay were significantly higher in the unfavorable outcome groups (*p* < 0.001) (Table 4).

**Figure 2:**
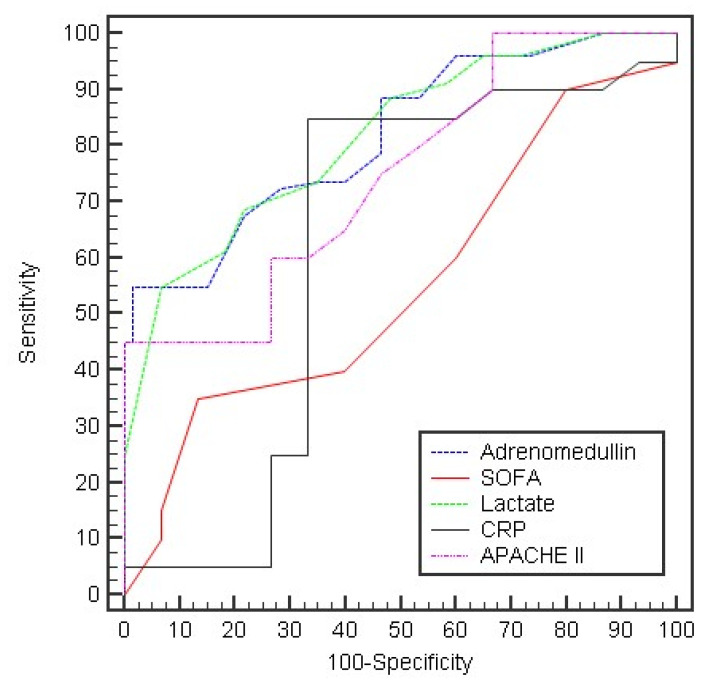
The ROC curve for the different parameters to predict outcome in day 0.

**Figure 3:**
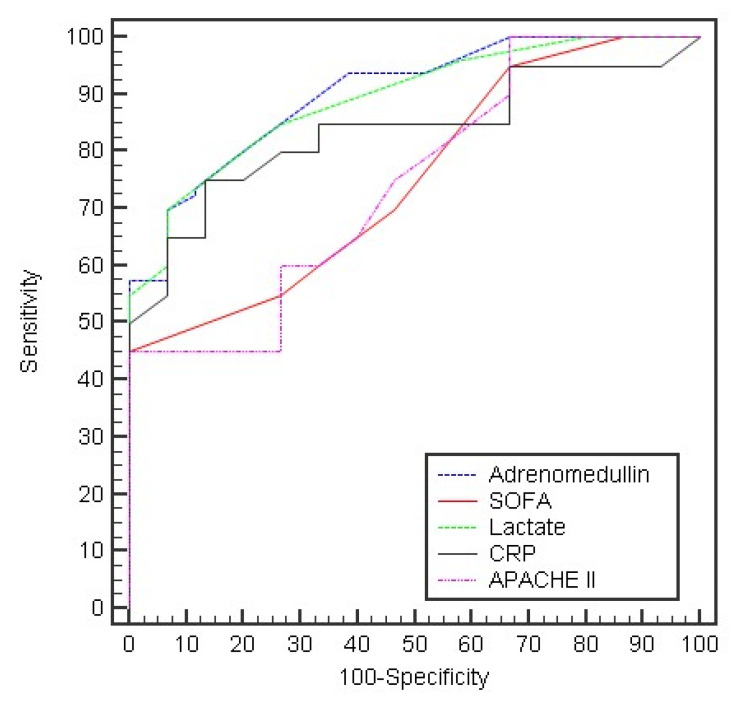
The ROC curve for different parameters to predict outcome in day 5.

The ROC curve of the different measured parameters was used to test the prediction of outcome at day 0. Only APACHE II, lactate, and adrenomedullin were statistically significant. Adrenomedullin showed the highest sensitivity (96.25%) with a cut-off value of >16 (AUC 0.818; 95% CI 0.751–0.885), while lactate showed the highest specificity (93.33%) with a cut-off value of >2.9 (AUC 0.817; 95% CI 0.749–0.885) (Table 5, Figure 2). The ROC curve at day 5 showed that all parameters were statistically significant. Adrenomedullin showed the highest specificity (100%) with a cut-off value of >20 (AUC 0.901; 95% CI 0.853–0.949), followed by lactate and CRP (specificity for both: 93.33%). Meanwhile, lactate showed the highest sensitivity (70.0%) (Table 5, Figure 3).

## 4. Discussion

Ventilator associated pneumonia as a common complication of mechanical ventilation heralds more ICU stay, mortality, and financial burden. A better understanding of risk factors for VAP is helpful in predicting the occurrence of VAP, improving the prevention and control of VAP, and reducing the morbidity and mortality rates of patients with VAP [16]. McEnery et al. [17] in their editorial reported that the independent risk factors identified as predictive for VAP were ICU length of stay, duration of ventilation, presence of tracheostomy, combined antibiotic use, APACHE score, frequency of oral care, and subglottic secretion management.

The Clinical Pulmonary Infection Score (CPIS) was used in the current study as a diagnostic tool. Its prognostic value was discouraged by Ghulam Khaleeq et al. [18]. According to the 2016 Clinical Practice Guidelines by the Infectious Diseases Society of America (IDSA) and the American Thoracic Society (ATS), for patients with suspected HAP/VAP, they suggest using the clinical criteria alone, rather than using CPIS plus clinical criteria to decide whether to initiate antibiotic therapy or not. They also suggested that endotracheal aspirate with semiquantitative cultures can be used to diagnose VAP, rather than invasive or noninvasive sampling with quantitative cultures (weak recommendation, low-quality evidence) [14]. In the present study, we combined modified CPIS (to incorporate tracheal aspirate with semiquantitative cultures) with the clinical criteria to diagnose VAP and to decide the initiating antibiotic therapy according to our local antibiotic policy, matching the guidelines of managing VAP [6,14].

In the current study, patients were statistically categorized according to their outcome distribution into favorable outcome (successful extubation) and unfavorable outcome (prolonged mechanical ventilation, persistent septic shock, and mortality). The role of ADM as one of the possible biomarkers in the prognosis of VAP was evaluated in comparison to some other parameters, namely, APACHE II score, SOFA score, lactate, and CRP, hoping to find out which of them could predict the incidence of unfavorable outcome (prolonged MV, septic shock, and mortality) at two-time intervals, namely, the day of confirmed VAP diagnosis and 5 days later.

The present study revealed that the age and sex of patients in all outcome groups was statistically insignificant (*p* = 0.061 and 0.555, respectively). APACHE II at days 0 and 5 was statistically significant, with the highest values belonging to patients who received prolonged mechanical ventilation, persistent septic shock, and mortality, denoting a statistical significance to predict unfavorable outcome (*p* < 0.001). X.-Y. Zhou et al. [19] in their study concluded that APACHE II score on admission was superior to CPIS for predicting 30-day mortality in patients with VAP. Gursel et al. [11] in their study concluded that only APACHE II >16 at the time of VAP diagnosis was an independent predictor of mortality.

SOFA score measurements at day 0 in the present study did not reach statistical significance differentiating favorable from unfavorable outcomes (*p =* 0.095). There was a significant statistical difference regarding SOFA score at day 5 differentiating patients with favorable from unfavorable outcomes (*p* < 0.001). However, it showed low sensitivity and specificity. Boeck et al. [20], in their study about SOFA score and copeptin for predicting survival in VAP, concluded that the predictive value of serial SOFA scores accurately predicted outcome in VAP patients. Núñez et al. [21], in their study on SOFA score performance and mortality risk factors in VAP patients with prolonged MV, found that the SOFA score was useful to predict fatal outcomes in such patients.

Madan et al. [12] revealed that there was a significant difference in the SOFA score on the day of diagnosing VAP between the survivors and non-survivors. Gursel et al. [11] showed that the mean SOFA score was significantly higher in the non-survivors compared to the survivors at the time of diagnosis of VAP despite no significant difference in the mean SOFA score between the survivors and non-survivors at the time of admission (*p* = 0.005). The discrepancy of these studies with the current work may be explained by the relatively short duration of MV before VAP diagnosis (about 3 days) in the current study. Regarding sepsis, Karakike et al. [22], in their analysis through a derivation and a validation cohort for early change in the SOFA score as a prognostic marker of 28-day sepsis mortality concluded that Δ SOFA on day 7 is a useful early prognostic marker of 28-day mortality.

Regarding the selected biomarkers, namely (CRP, lactate, and adrenomedullin) in the current study that were obtained at day 0 and day 5, lactate and ADM were the only early predictors of occurrence of unfavorable outcome at day 0 (together with APACHI II score). ADM showed a superiority over lactate in predicting unfavorable outcome with a cut-off value of 16 pg./mL. It showed a high sensitivity (96.25%), but with low specificity (40%). Day 5 readings of all of the measured biomarkers, in addition to the APACHI II and SOFA scores, could predict an unfavorable outcome. ADM had the highest specificity (100%) but with low sensitivity (57.5%). Lactate showed a sensitivity and specificity of 70% and 93.3%, respectively.

Póvoa et al. [23], in their pilot study on 47 confirmed VAP patients, concluded that the pattern of CRP response after 4 days of treating VAP was useful in the identification of poor outcome. Ehlenz K et al. [24] concluded that the plasma ADM levels were elevated not only in sepsis, but also in cancer patients and shock state with impaired renal excretion. When ADM was correlated with the C-reactive protein levels in these patients, it could identify sepsis or cancer patients. Hillas et al. [25], in their study, evaluated the performance of procalcitonin and CRP as predictors of poor outcome in 45 VAP patients. They noticed significantly high levels of CRP and procalcitonin at different time intervals in those complicated by septic shock, but neither of both markers could predict the VAP survival or deterioration to septic shock.

Caironi P et al. [26], in their study on 956 patients with sepsis and septic shock, found that serum biologically active ADM (bio-ADM) may help tailor hemodynamic support therapy and can predict 90-day mortality. Plasma bio-ADM on day 1 was higher in patients with septic shock, multiple organ failure, and serum lactate curve over the first week, giving clues about the association between high lactate, bio-ADM, and the prediction of poor outcome in such patients. Bou Chebl et al. [27], in their retrospective study on 450 critically ill patients presented to the Emergency Department (ED), found that high lactate values were associated with higher mortalities and longer ED and hospital stays. This was in favor of the predictive ability of higher lactate levels on poor outcome in the critically ill. Lee et al. [28] stated that septic shock can be diagnosed under two conditions: persistent hypotension despite adequate resuscitation and serum lactate level >2 mmol/L. They reported that the mortality rate of patients with lactate ≥4 mmol/L was 30% and if combined with hypotension, the mortality rate reached 46.1%.

Chen et al. [9] conducted a study on 372 consecutive septic patients admitted to the emergency department. They concluded that ADM was the only independent predictor of the development of severe sepsis and septic shock in those patients. They found that ADM sensitivity was 67.6% and the specificity was 90.0%. However, they did not evaluate the role of ADM in predicting mortality. Tamer et al. [10], in their work about the prognostic role of ADM in sepsis, concluded that admitting serum adrenomedullin in septic patients may be an early predictor of unfavorable outcome. They measured the deterioration to septic shock and 28-day mortality as the unfavorable outcome. Marino R et al. [29], in their prospective analysis of 101 patients presented to the emergency department with suspected sepsis, concluded that ADM is strongly associated with the severity of disease, vasopressor requirement, and 28-day mortality.

There are some limitations to be considered in this study. The main limitation is the lack of predictive model development. The studied parameters were significantly correlated either at day 0, day 5, or even both, and a strong correlation was found between ADM and the other studied parameters. This would create a problem of multicollinearity if regression analysis is conducted. Furthermore, this study excluded a large sector of patients, namely, renal, hepatic, and most cardiac patients due to the direct or indirect influence on the ADM level. This can make serum ADM not a suitable marker alone. Comparing frequent successive samples of ADM for the same patient may help overcome such a problem. In addition, the lack of solid and uniform VAP diagnostic criteria is the limitation of all studies on ventilator associated pneumonia.

## 5. Conclusions

Serum adrenomedullin when measured at day 0 of VAP diagnosis may serve as an early predictor of unfavorable outcome with a cut-off value of 16 pg./mL (sensitivity: 96.25% and specificity: 40%). When repeated at day 5, ADM with a cut-off value of 20 pg./mL showed a sensitivity of 57.5% and specificity of 100% to predict the prolonged mechanical ventilation, septic shock, and mortality in VAP patients.

## Figures and Tables

**Table 1:** The baseline patients’ criteria at the time of enrollment.

	Extubation (*n* = 60)	Prolonged MV (*n* = 28)	Septic Shock (*n* = 20)	Died (*n* = 32)	χ^2^	*p*
**Age (years):** Range Mean ± SD	19–86	28–70	50–66	51–73	(F) =	0.061
	51.3 ± 24.6	50.1 ± 14.9	58.0 ± 6.22	60.4 ± 7.8	2.515	
**Sex:** Male (No%) Female (No%)	32 (53.3)	16 (57.1)	12 (60.0)	22 (68.8)	2.085	0.555
	28 (46.7)	12 (42.9)	8 (40.0)	10 (31.3)		
**Comorbidities:** DM (%) HTN (%) IHD (%) COPD (%)	44 (73.3)	20 (71.4)	15 (75.0)	23 (71.86)	2.101	0.989
	38 (63.3)	17 (60.7)	12 (60.0)	17 (53.13)		
	27 (45.0)	12 (42.9)	8 (40.0)	13 (40.63)		
	12 (20.0)	7 (25.0)	3 (15.0)	3 (9.38)		
**MV days before VAP** Range Mean ± SD	2–5	3–5	2–6	2–4	(F) = 2.703	0.641
	3.1 ± 0.98	2.58 ± 1.03	2.06 ± 1.9	3.06 ± 0.08		
**Main diagnosis:** Coma (%) Respiratory failure (%) Sepsis (%) Toxidromes (%)	31 (51.67)	12 (42.86)	11 (55.0)	18 (56.25)	2.246	0.989
	12 (20.0)	7 (25.0)	3 (15.0)	5 (15.63)		
	12 (20.0)	5 (17.86)	4 (20.0)	6 (18.75)		
	5 (8.3)	4 (14.29)	2 (10.0)	3 (9.38)		
**Culture of Tracheal Aspirate at day 0:** MRSA (%) Pseudomonas (%) Klebsiella (%) E-coli (%) Acinetobacter (%) Candida (%) Contaminated (%)	4 (6.7)	0 (0.0)	0 (0.0)	0 (0.0)	4.015	0.999
	0 (0.0)	0 (0.0)	4 (14.3)	0 (0.0)		
	20 (33.3)	20 (71.4)	12 (42.8)	24 (75.0)		
	4 (6.7)	0 (0.0)	0 (0.0)	4 (12.5)		
	32 (53.3)	8 (28.6)	12 (42.8)	4 (12.5)		
	0 (0.0)	0 (0.0)	0 (0.0)	4 (12.5)		
	4 (6.7)	0 (0.0)	0 (0.0)	0 (0.0)		

Values are presented as mean ± standard deviation (SD), number (*n*), and percentage (%). MV—mechanical ventilation. VAP—ventilator associated pneumonia, DM—diabetes mellitus, HTN—hypertension, IHD—ischemic heart disease, COPD—chronic obstructive pulmonary disease, Day 0—day of confirmed VAP diagnosis. χ^2^—Chi-square test. *P*—probability for comparing between both groups.

**Table 2:** The patients’ distribution according to the different parameters at different intervals.

Parameter:	Time Interval	Extubation (*n* = 60)	Prolonged MV (*n* = 28)	Septic Shock (*n* = 20)	Died (*n* = 32)	*F/H*	*p*
**APACHE II** (Mean ± SD)	**Day 0**	11.1 ± 4.6	12.9 ± 3.1	17.8 ± 5.4	17.1 ± 4.4	19.316 *	<0.001 *
pExtupation			0.323	<0.001 *	<0.001 *		
Sig. bet. Grps	p1 = 0.001 *, p2 = 0. 002 *, p3 = 0.950						
(Mean ± SD) pExtupation Sig. bet. Grps	**Day 5**	10.9 ±3.9	13.6 ± 2.9	21.3 ± 3.2	20.6 ± 2.9	20.932 *	<0.001 *
			0.291	<0.001 *	<0.001 *		
	p1 < 0.001 *, p2 = 0.001 *, p3 = 0.694						
**CRP (mg/L)**							
(Mean ± SD)	**Day 0**	37.3 ± 33.7	45.7 ± 8.5	44.0 ± 44.4	44.6 ± 14.7	0.806	0.493
(Mean ± SD)	**Day 5**	18.6 ± 7.54	28.3 ± 9.5	52.0 ± 19.2	34.4 ± 15.8	59.320 *	<0.001 *
pExtupation			<0.001 *	<0.001 *	<0.001 *		
Sig. bet. Grps	p1 < 0.001 *, p2 = 0.300, p3 = 0.006 *						
**Lactate (mmol/L)**							
(Mean ± SD)	**Day 0**	2.39 ± 0.38	2.75 ± 0.28	2.78 ± 0.42	2.97 ± 0.30	21.535 *	<0.001 *
pExtupation			<0.001 *	<0.001 *	<0.001 *		
Sig. bet. Grps	p1 = 0.994, p2 = 0.235, p3 = 0.235						
(Mean ± SD)	**Day 5**	2.08 ± 0.35	2.7 ± 0.54	3.64 ± 0.89	3.33 ± 0.66	49.363*	< 0.001*
pExtupation			<0.001 *	<0.001 *	<0.001 *		
Sig. bet. Grps	p1 < 0.001 *, p2 < 0.001 *, p3 = 0.855						
**SOFA score**							
(Mean ± SD)	**Day 0**	6.07 ± 1.62	6.7 ± 2.16	7.0 ± 2.25	5.88 ± 1.29	6.368	0.095
(Mean ± SD)	**Day 5**	3.60 ± 1.80	4.29 ± 1.86	6.60 ± 2.64	6.38 ± 2.03	41.485 *	<0.001 *
pExtupation			<0.001 *	<0.001 *	<0.001 *		
Sig. bet. Grps	p1 = 0.001 *, p2 = 0.001 *, p3 = 0.892						
**Adrenomedullin (pg/mL)**							
(Mean ± SD)	**Day 0**	19.1 ± 8.42	34.1 ± 18.2	42.4 ± 26.7	49.8 ± 23.0	46.703*	<0.001 *
pExtupation			<0.001 *	<0.001 *	<0.001 *		
Sig. bet. Grps	p1 = 0.579, p2 = 0.027*, p3 = 0.149						
(Mean ± SD)	**Day 5**	7.82 ± 5.16	18.5 ± 9.66	52.5 ± 35.2	43.9 ± 29.1	74.411 *	<0.001 *
pExtupation			<0.001 *	<0.001 *	<0.001 *		
Sig. bet. Grps	p1 = 0.016 *, p2 = 0.010 *, p3 = 0.897						

Values are presented as mean ± standard deviation (SD), number (*n*). Sig. bet. Grps—significance between groups, F and H values for ANOVA and Kruskal–Wallis tests, respectively, *p*—pairwise comparison between each two groups was conducted using the post hoc test (Dunn’s test for multiple comparisons test), pExtupation—*p* value for comparison between extubation and each other group, p1—comparing between prolonged MV and septic shock. p2—comparison between the prolonged MV and died. p3—comparison between septic shock and died. * Significant differences from the baseline *p* ≤ 0.05. Day 0—day of confirmed VAP diagnosis. Day 5—5 days later.

**Table 3:** The correlation between adrenomedullin and other parameters at different intervals.

Parameter:	Adrenomedullin (ADM)			
	Day 0		Day 5	
	*r_s_*	*p*	*r_s_*	*p*
**CRP**	0.085 ^$^	0.320	0.702 ^$^	<0.001 *
**SOFA score**	0.262 ^#^	0.049 *	0.820 ^#^	<0.001 *
**APACHE II score**	0.437 ^#^	<0.001 *	0.814 ^#^	<0.001 *
**Lactate**	0.992 ^$^	<0.001 *	0.990 ^$^	<0.001 *

^#^ Correlation coefficient for the Pearson correlation, ^$^ Correlation coefficient for the Spearman rank correlation, * Statistically significant at *p* ≤ 0.05. Day 0—day of confirmed VAP diagnosis. Day 5—5 days later.

**Table 4:** The patients’ distribution according to mechanical ventilation days and ICU stay.

	Extubation (*n* = 60)	Prolonged M.V (*n* = 28)	Septic Shock (*n* = 20)	Died (*n* = 32)	*F*	*p*
**M.V (days)**						
(Mean ± SD)	8.6 ± 1.09	14.4 ± 1.20	16.6 ± 1.90	14.38 ± 1.90	227.81 *	<0.001 *
pExtupation		<0.001 *	<0.001 *	<0.001 *		
Sig. bet. Grps	p1 < 0.001 *, p2 = 0.999, p3 < 0.001 *					
**ICU stay (days)**						
(Mean ± SD)	11.13 ± 0.72	18.7 ± 1.5	20.8 ± 1.77	14.38 ± 1.90	337.6 *	<0.001 *
pExtupation		<0.001 *	<0.001 *	<0.001 *		
Sig. bet. Grps	p1 < 0.001 *, p2 < 0.001 *, p3 < 0.001 *					

Values are presented as mean ± standard deviation (SD), M.V—mechanical ventilation, Sig. bet. Grps—significance between groups, *F* and *p* values for ANOVA test. Pairwise comparison between each two groups was conducted using the post hoc test (Dunn’s test for multiple comparisons test). pExtupation—*p* value for comparing between extubation and each other group. *p*1—comparison between prolonged MV and septic shock. *p*2—comparison between prolonged MV and died. *p*3—comparison between septic shock and died. * Significant differences from baseline *p* ≤ 0.05.

**Table 5:** The areas under the receiver operating characteristic curves to predict outcome in days 0 and 5.

Day 0:	AUC	*p*	95% C.I	Cut off	Sensitivity	Specificity	PPV	NPV
**Adrenomedullin**	0.818 *	<0.001 *	0.751–0.885	>16	96.25	40.0	68.1	88.9
**SOFA**	0.555	0.266	0.459–0.651	≤5	50.0	60.0	62.5	47.4
**Lactate**	0.817 *	<0.001 *	0.749–0.885	>2.9	55.0	93.33	91.7	60.9
**CRP**	0.552	0.296	0.450–0.654	>22	60.0	66.67	70.6	55.6
**APACHE II**	0.747 *	<0.001 *	0.667–0.826	>12	65.0	60.0	68.4	56.3
**Day 5:**								
**Adrenomedullin**	0.901 *	<0.001 *	0.853–0.949	>20	57.50	100.0	100.0	63.8
**SOFA** **Lactate** **CRP** **APACHE II**	0.752 * 0.893 * 0.835 * 0.747 *	<0.001 * <0.001 * <0.001 * <0.001 *	0.673–0.830 0.842–0.943	>5 >2.5	55.0 70.0	73.33 93.33	73.3 93.3	55.0 70.0
			0.768–0.902 0.667–0.826	>34 >12	55.0 65.0	93.33 60.0	91.7 68.4	60.9 56.3

C.I—confidence interval, NPV—negative predictive value, PPV—positive predictive value, * Statistically significant at *p* ≤ 0.05. Day 0—day of confirmed VAP diagnosis. Day 5—5 days later.
